# Social Fragility: Impact of Early Fertility and Domestic Violence in Colombia During the Pandemic

**DOI:** 10.3390/ijerph22030453

**Published:** 2025-03-20

**Authors:** Fabian Dávila, Favio Cala-Vitery

**Affiliations:** Public Policy Modeling and Management, School of Natural Sciences and Engineering, Universidad de Bogotá Jorge Tadeo Lozano, Bogotá 111711, Colombia; favio.cala@utadeo.edu.co

**Keywords:** early motherhood, inequality, gender violence, cluster analysis, public policies

## Abstract

Social fragility, defined as the inability of a society to manage risks and resolve conflicts without violence or external aid, is closely linked to early fertility and domestic violence, particularly among vulnerable populations. These challenges were exacerbated by the COVID-19 pandemic, which exposed regional disparities across Colombia, particularly in areas with weak social infrastructure and high dependence on public policies. This study integrates data from all Colombian departments on annual births, deaths by cause, domestic violence by perpetrator, and other demographic factors to construct key indicators of early fertility and structural inequalities. To measure fragility, we developed the Early Fertility Fragility Index (EFFI) and refined it using a Generalized Linear Model (GLM) to identify the most critical predictors of early fertility risk. The findings reveal marked regional disparities, with departments exhibiting high Indigenous and migrant populations, high domestic violence rates, and weak social protection systems experiencing the greatest fragility and the most pronounced increases in early fertility. However, these results reflect correlational relationships rather than causal effects. Further research using longitudinal or experimental designs is needed to establish causality.. In contrast, urbanized regions with stronger social and economic infrastructures showed greater resilience. These results highlight how structural inequalities intensify the effects of crises on vulnerable populations. The refined EFFI model provides a robust framework for assessing regional fragility and guiding evidence-based policy interventions. Addressing these disparities requires regionally tailored strategies that prioritize investment in social infrastructure, reproductive health services, and protective policies to mitigate the long-term consequences of early fertility and social fragility in Colombia.

## 1. Introduction

Social fragility, defined as the inability of a system to manage risks and guarantee essential services autonomously, is a critical challenge in regions marked by deep structural inequalities [1]. These inequities exacerbate vulnerabilities, reinforcing cycles of exclusion that hinder access to essential services and limit the effectiveness of public policies [2]. In contexts where territorial fragmentation and economic disparities prevail, fragile systems struggle to address the needs of marginalized populations, further entrenching social instability and dependency on state interventions [3]. Recent studies emphasize the role of resilience frameworks in mitigating the adverse effects of social fragility, highlighting the need for multidimensional approaches to assess and address these vulnerabilities [4,5].

The COVID-19 pandemic intensified pre-existing vulnerabilities, such as limited access to reproductive health services. Additionally, it introduced new challenges, including disruptions in family planning programs, mobility restrictions, and increased household confinement, which further exacerbated early fertility rates and domestic violence. Disruptions in family planning programs, mobility restrictions, and socioeconomic instability led to rising early fertility rates, particularly in underserved communities [6,7]. Additionally, socioeconomic instability, job losses, and prolonged exposure to domestic confinement heightened stressors for vulnerable groups, leading to increased incidences of gender-based and domestic violence [8]. Such disparities align with global patterns observed in crisis response and resilience-building efforts [9].

A systematic review by Dávila et al. (2025) [10], identified key determinants influencing adolescent girls’ access to SRH services in Latin America, including economic barriers, regulatory restrictions, institutional capacity, and social stigma. These findings informed the selection of risk-related indicators used in this study to construct the Early Fertility Fragility Index (EFFI). The EFFI integrates structural inequality, early fertility, domestic violence, and violent deaths as core dimensions of fragility, providing a comprehensive measure of regional vulnerability. Recent studies underscore the importance of incorporating resilience-based models to assess the interplay of economic, social, and institutional factors in crisis response [4,5].

To refine the EFFI definition and assess its predictive capacity, this study employs a Generalized Linear Model (GLM) approach, identifying the most significant risk factors influencing early fertility. By analyzing departmental-level variations in fertility trends and social fragility, the study provides a robust framework for evaluating regional disparities. The findings contribute to evidence-based policymaking, supporting the design of targeted interventions aimed at mitigating early fertility risks and strengthening social resilience in Colombia.

## 2. Materials and Methods

This study integrates data from three public sources at the departmental and annual levels, ensuring comparability across temporal and geographic dimensions:

Deaths and Births: National Administrative Department of Statistics (DANE) dataset, disaggregated by maternal age, cause of death, and date of occurrence [11].

Health Insurance: Ministry of Social Protection records on social security affiliation, categorized by regime, age, and department of residence [12].

Domestic Violence: Open data from the National Institute of Legal Medicine and Forensic Sciences, detailing violence reports by age, gender, and event characteristics [13].

All data was exported in CSV format and harmonized, ensuring consistency across departments and years. Beyond imputation using linear interpolation, we applied normalization techniques (Z-score transformation), conducted cross-source validation, and standardized variable definitions to ensure comparability across datasets.

The selection of indicators for defining fertility-related fragility is based on prior research by Dávila et al. (2025), which identified key structural determinants affecting adolescent reproductive health in Latin America [10]. These indicators were chosen due to their strong association with early fertility outcomes, as demonstrated in previous empirical studies. The Fertility Fragility Index (FFI) captures four dimensions of vulnerability:

Structural Inequality: Measures demographic and socioeconomic disparities affecting reproductive health access:

Percentage of Indigenous Affiliated Women (*%PIW*):

$$\%PIW=\left(\frac{Indigenous\;Affiliated\;Women}{Total\;Affiliated\;Women}\right)\times 100$$

Percentage of Migrant Affiliated Women (*%PMW*):

$$\%PMW=\left(\frac{Migrant\;Affiliated\;Women}{Total\;Affiliated\;Women}\right)\times 100$$

Percentage of Subsidized Affiliated Women *(%PSW):*

$$\%PSW=\left(\frac{Subsidized\;Affiliated\;Women}{Total\;Affiliated\;Women}\right)\times 100$$

Early fertility: Defined from previous research [10,14,15] by maternal age categories:

Infant Fertility Rate (*IFR*):

$$IFR=\left(\frac{\sum Births\;from\;Mothers\;\leq 14}{\sum Affiliated\;Women\;10-14\;}\right)\times 1000$$

Adolescent Fertility Rate (*AFR*):

$$AFR=\left(\frac{\sum Births\;from\;Mothers\;15-19\;}{\sum Affiliated\;Woman\;15-19}\right)\times 1000$$

Early Fertility Rate (*EFR*):

$$EFR=\left(\frac{\sum Births\;from\;Mothers\;\leq 19}{\sum Affiliated\;Woman\;10-19}\right)\times 1000$$

Domestic violence: Indicators reflecting violence exposure affecting adolescent health:

Percentage of Domestic Violence Against Children (*DVAC*):

$$\%DVAC=\left(\frac{Cases\;of\;Domestic\;Violence\;Against\;Children\leq 18\;}{Total\;cases\;of\;Domestic\;violence\;reported}\right)\times 100$$

Percentage of Domestic violence against women (*DVAW*) from a male perpetrator:

$$\%DVAW=\left(\frac{Cases\;of\;Domestic\;Violence\;Against\;Woman}{Total\;cases\;of\;Domestic\;violence\;reported}\right)\times 100$$

Violent Deaths: Reflects lethal violence risks among adolescents.

Suicide Rate (*SR*):

$$SR=\left(\frac{Death\;cases\;by\;suicide}{Total\;deaths\;reported}\right)\times 1000$$

Child Homicide Rate (*CHR*):

$$CHR=\left(\frac{Homicide\;cases\;in\;\leq 19\;Years}{Total\;deaths\;reported}\right)\times 1000$$

Construction of the Fertility Fragility Index (FFI): The Fertility Fragility Index (FFI) is a composite measure designed to capture multidimensional risks related to early fertility, focusing on structural vulnerabilities and exposure to risk factors rather than just fertility outcomes.

Step 1: Normalization of Indicators: Each indicator was standardized using Z-score transformation. This method was chosen because it preserves the distribution of the original data and ensures comparability between variables measured in different units, unlike min-max scaling which compresses values into a fixed range:

$$Z_{i}=\frac{X_{i}-\mu }{\sigma }$$

where *X*_*i*_ is the original indicator value, *μ* is the mean, and *σ* is the standard deviation.

Step 2: Selection of Indicators Measuring Fertility Fragility Causes: To measure fertility fragility, we focus on structural and social determinants that increase adolescent fertility risk, rather than the direct change in fertility rates.

Structural Inequality Indicators (Barriers to Reproductive Health Access & Social Vulnerability):

*Z_PIW,d_* = Standardized percentage of Indigenous Affiliated Women (reflecting socioeconomic exclusion).

*Z_PMW,d_* = Standardized percentage of Migrant Affiliated Women (capturing mobility and lack of service continuity).

*Z_PSW,d_* = Standardized percentage of Subsidized Affiliated Women (proxy for economic vulnerability).

Exposure to Risk Factors (Social Conditions Increasing Fertility Fragility & Vulnerability):

*Z_DVAW,d_* = Standardized percentage of Domestic Violence Against Women (reproductive autonomy barriers).

*Z_DVAC,dZ_* = Standardized percentage of Domestic Violence Against Children (early exposure to trauma).

*Z_CHR,dZ_* = Standardized Child Homicide Rate (capturing extreme social instability).

Step 3: To construct and refine the Early Fertility Fragility Index (EFFI), the analysis was based on a segmentation of data into two distinct datasets:

Longitudinal dataset: This dataset included annual records of risk-related indicators, explicitly excluding early fertility indicators to prevent issues of collinearity. This structure allowed for tracking changes in structural vulnerabilities and risk factors over time across different departments.

Aggregated dataset: This dataset was used to calculate the outcome variable (EFFI), focusing on the change in early fertility rates in the first post-pandemic year. The approach is based on the hypothesis that pregnancies initiated during the pandemic (2020) resulted in maternities in 2021, making it possible to assess the impact of the health crisis on early fertility trends.

By integrating these datasets, the model ensures a clearer assessment of the relationship between structural risk factors and changes in early fertility rates while avoiding redundancy in explanatory variables. This segmentation also enhances the robustness of the GLM model, ensuring that the predictors remain independent and do not confound the analysis of fertility dynamics.

Step 4: Aggregation of Indicators into FFI: The final Fertility Fragility Index (FFI) was calculated after removing non-significant variables. Variables were excluded if their *p*-values exceeded 0.05 in the GLM or if they did not significantly improve model fit, as measured by pseudo-R^2^ and deviance reduction to ensure a more precise aggregation of risk-related indicators. This refinement process enhances the robustness of the index by including only the most relevant predictors, allowing for a more accurate representation of structural vulnerabilities associated with early fertility.

$$FFId=w_{1}Z_{PIW,d}+w_{2}Z_{PMW,d}+w_{3}Z_{PSW,d}+w_{4}Z_{DVAW,d}+w_{5}Z_{DVAC,d}+w_{6}Z_{CHR,d}$$

where: $Z_{i,d}$ represents the standardized value of indicator *i* in department *d* and $w_{i}$ is the weight assigned to each indicator.

Step 5: Validation of the Fertility Fragility Index (FFI): Since FFI is designed to measure fragility risks, we expect that departments with higher fragility scores will exhibit larger changes in early fertility rates. To test this, we computed Spearman’s rank correlation between the FFI and the relative annual percentage changes in fertility rates.

Relative Change in Infant Fertility Rate (Δ*IFR*):

$$\Delta IFR=\left(\frac{{IFR}_{t}-{IFR}_{t-1}}{{IFR}_{t-1}}\right)\times 100$$

Relative Change in Adolescent Fertility Rate (Δ*AFR*):

$$\Delta AFR=\left(\frac{{AFR}_{t}-{AFR}_{t-1}}{{AFR}_{t-1}}\right)\times 100$$

Relative Change in Early Fertility Rate (Δ*EFR*):

$$\Delta EFR=\left(\frac{{EFR}_{t}-{EFR}_{t-1}}{{EFR}_{t-1}}\right)\times 100$$

Spearman’s correlation coefficient (*ρ*) measures the monotonic relationship between FFI and fertility changes, testing whether higher fragility scores correspond to higher fertility rate changes.

Expected Results: *ρ* > 0 (positive correlation): Suggests that higher fragility scores are associated with greater increases in early fertility, supporting the validity of the FFI; *ρ* ≈ 0 (no correlation): Indicates that fragility scores are not directly linked to fertility changes, suggesting that other factors may be influencing fertility trends; and *ρ* < 0 (negative correlation): Would be unexpected, as it would suggest that higher fragility is linked to lower fertility rate increases, contradicting the theoretical framework.

The results are presented through descriptive and graphical methods to illustrate early fertility fragility patterns and validate the Early Fertility Fragility Index (EFFI). Frequency distribution tables summarize central tendencies and variability across departments, providing a clear overview of EFFI indicators. Scatter plots with trend lines and confidence intervals explore the associations between the Early Fertility Fragility Index (EFFI) and early fertility indicators. Heatmaps visualize geographical disparities in fragility indicators, allowing for regional comparisons of vulnerability. Finally, Spearman correlation analyses are displayed in both tabular and graphical formats, assessing the relationship between the EFFI and fertility rate changes.

Data extraction and harmonization were conducted using Microsoft Excel Power Query. Statistical analysis and visualization were performed using Python 3 (Google Colab), employing Pandas and NumPy for data manipulation and computation of standardized indicators. Matplotlib 3.10.0 and Seaborn 0.13.2 were used for generating scatter plots, heat maps, and other visualizations. SciPy was used to compute Spearman correlation coefficients to validate the FFI. The analytical workflow was designed to ensure reproducibility, maintaining consistency across data processing, indicator computation, and validation steps. Detailed scripts and Supplementary Materials are available for further reference.

## 3. Results

Table 1 presents the structural inequality indicators across Colombian departments from 2019 to 2022, including the percentage of Indigenous-affiliated women (%PIW), migrant-affiliated women (%PMW), and subsidized affiliated women (%PSW). The highest median %PIW values were observed in Vaupés (74.43%), Guainía (60.94%), and La Guajira (43.31%), reflecting a strong presence of Indigenous populations in these regions, urbanized areas such as Bogotá D.C. (0.08%) and San Andrés (0%) reported minimal Indigenous affiliation. Variability was most pronounced in Guainía (52.53–69.51%), suggesting shifts in indigenous enrollment over time.

The highest %DVAW was recorded in Chocó (403.27), Sucre (376.09), and Córdoba (369.83), indicating significantly higher rates of reported domestic violence cases per 1000 total cases. In contrast, the lowest median %DVAW values were observed in Guaviare (257.81) and Vaupés (263.16), though with high variability in some regions (e.g., Guaviare: 0–777.78), suggesting sporadic but extreme instances of domestic violence reporting.

For domestic %DVAC, Vichada (735.29) exhibited the highest median, with large variability (230.77–1000). Other departments with high median values included Amazonas (144.32) and Guaviare (115.83). Nariño (47.65) and Cauca (58.17) reported the lowest %DVAC.

The CHR was particularly high in Chocó (24.76 per 1000 deaths), San Andrés (17.74 per 1000 deaths), and Putumayo (11.10 per 1000 deaths), underscoring critical security concerns in these departments. The lowest CHR values were found in Boyacá (1.27 per 1000 deaths) and Bogotá D.C. (1.87 per 1000 deaths), suggesting relatively lower risks of child homicide in these urbanized regions (See Table 2).

The highest median IFR was observed in Vichada (5.07 per 1000 girls aged 10–14), Guainía (4.64 per 1000), and Guaviare (2.85 per 1000), indicating significant early fertility risks in these regions. In contrast, the lowest IFR was found in San Andrés (0.24 per 1000), Bogotá D.C. (0.34 per 1000), and Boyacá (0.62 per 1000); for AFR, the highest median values were reported in Vichada (70.63 per 1000 adolescents aged 15–19), Guainía (51.51 per 1000), and La Guajira (49.21 per 1000), highlighting elevated adolescent fertility risks. Conversely, the lowest AFR values were seen in Valle del Cauca (21.17 per 1000), San Andrés (21.21 per 1000), and Bogotá D.C. (14.82 per 1000). The Early Fertility Rate (EFR), which reflects the overall fertility burden among girls aged 10–19, followed similar patterns. Vichada (36.5 per 1000), Guainía (26.76 per 1000), and La Guajira (25.08 per 1000) exhibited the highest risks, while Bogotá D.C. (7.63 per 1000), San Andrés (10.63 per 1000), and Boyacá (11.00 per 1000) had the lowest early fertility rates (See Table 3).

**Figure 1:**
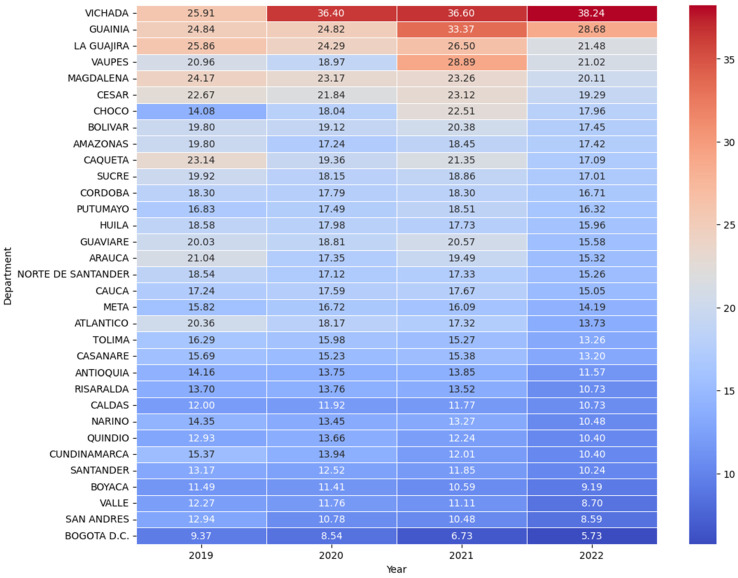
Heatmap sorted by Early Fertility Rate (EFR) and Year (Colombia 2019 to 2022). This heatmap illustrates the evolution of Early Fertility Rate (EFR) across Colombian departments from 2019 to 2022. The Departments were sorted by EFR for the most recent year (2022) to highlight those with persistently high or shifting fertility patterns over time. The color gradient reflects changes in fertility levels, with warmer tones indicating higher EFR values and cooler tones representing lower rates.

Regions with consistently high EFR over time include Vichada, Guainía, La Guajira, and Vaupés, maintaining rates above 20 births per 1000 girls aged 10–19 across all years. Vichada exhibited the highest EFR values, increasing from 25.91 in 2019 to 38.24 in 2022, indicating an upward trend. Similarly, Guainía showed fluctuations but remained among the highest, peaking at 33.37 in 2021. In contrast, Bogotá D.C., San Andrés, Valle del Cauca, and Boyacá consistently reported the lowest EFR values, with Bogotá D.C. experiencing a steady decline from 9.37 in 2019 to 5.73 in 2022. A downward trend is also observed in Atlántico, Tolima, Antioquia, Risaralda, and Caldas, reflecting a gradual reduction in early fertility rates over time (See Figure 1).

**Figure 2:**
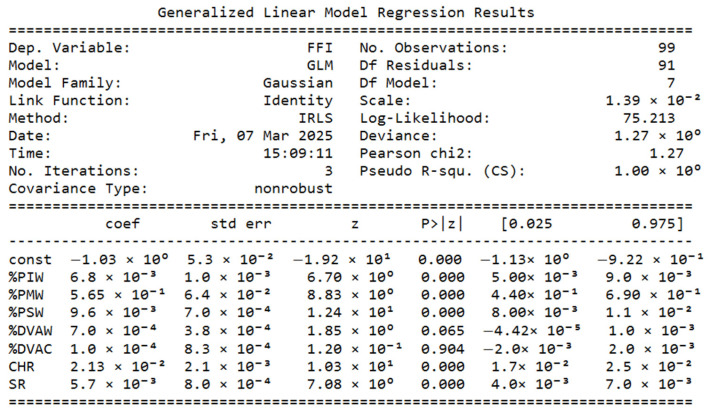
Generalized Linear Model Regression for FII vs. Risk indicators (1st model).

The GLM results indicate that migrant affiliation (%PMW), child homicide rate (CHR), and subsidized health affiliation (%PSW) are the strongest predictors of Early Fertility Fragility Index (EFFI) (*p* < 0.001). Additionally, Indigenous affiliation (%PIW) and suicide rate (SR) also show significant associations, suggesting that structural vulnerabilities contribute to early fertility risks. In contrast, domestic violence against children (%DVAC) and women (%DVAW) were not significant predictors. The model explains a substantial portion of the variance (pseudo R^2^ = 1.000, deviance = 1.2684), reinforcing that EFFI effectively captures fragility dimensions linked to early fertility (See Figure 2).

**Figure 3:**
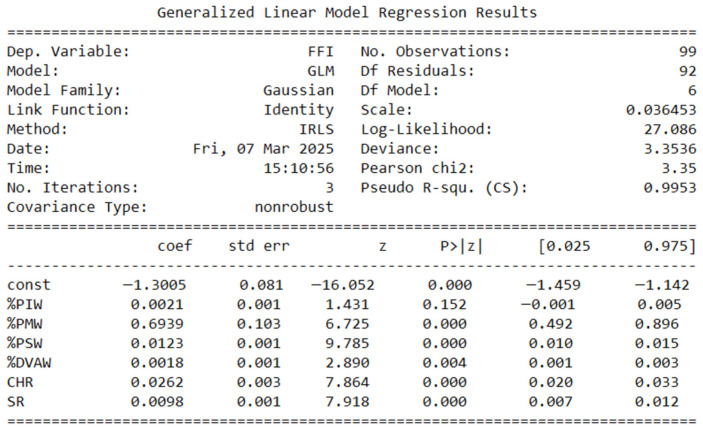
Generalized Linear Model Regression for FII vs. Risk indicators (2nd model).

After removing non-significant predictors, the refined GLM model maintains a strong explanatory power (pseudo R^2^ = 0.9953, deviance = 3.3536), confirming the robustness of the Early Fertility Fragility Index (EFFI). Migrant affiliation (%PMW), subsidized health affiliation (%PSW), child homicide rate (CHR), and suicide rate (SR) remain highly significant (*p* < 0.001), reinforcing their role in early fertility vulnerability. Domestic violence against women (%DVAW) also emerged as a significant predictor (*p* = 0.004), suggesting its influence on fragility. Although Indigenous affiliation (%PIW) was retained in the model, its effect was not statistically significant (*p* = 0.152), indicating a weaker contribution (Figure 3).

**Figure 4:**
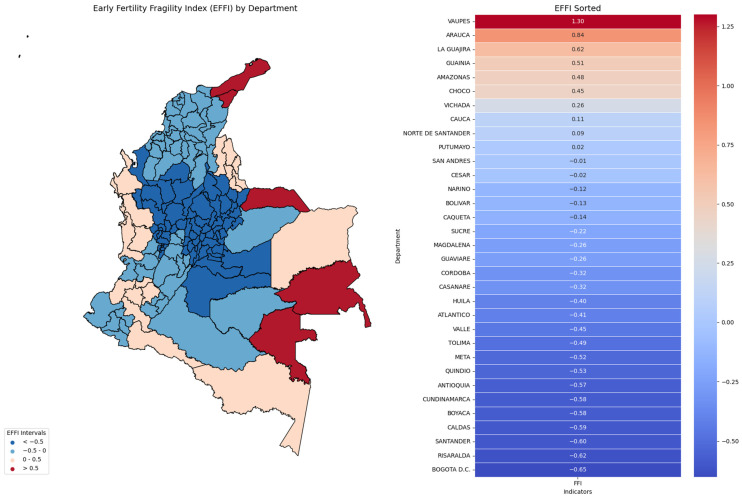
Early Fertility Fragility Index (EFFI) by Department in Colombia (2019–2021). This figure presents the spatial distribution of the EFFI across Colombian departments. The left panel shows a choropleth map with fragility levels categorized into four intervals, highlighting regional disparities. The right panel ranks departments by EFFI using a heatmap, emphasizing the contrast between high and low-fragility areas.

The highest EFFI values were observed in Arauca (0.87), La Guajira (0.66), and Guainía (0.47), followed by Vichada (0.45), Chocó (0.42), and Amazonas (0.33). In contrast, the lowest EFFI values were found in Caldas (−0.63), Risaralda (−0.62), Santander (−0.57), Bogotá D.C. (−0.57), and Boyacá (−0.56). These differences highlight spatial heterogeneity in the factors contributing to early fertility fragility across departments (See Figure 4).

**Figure 5:**
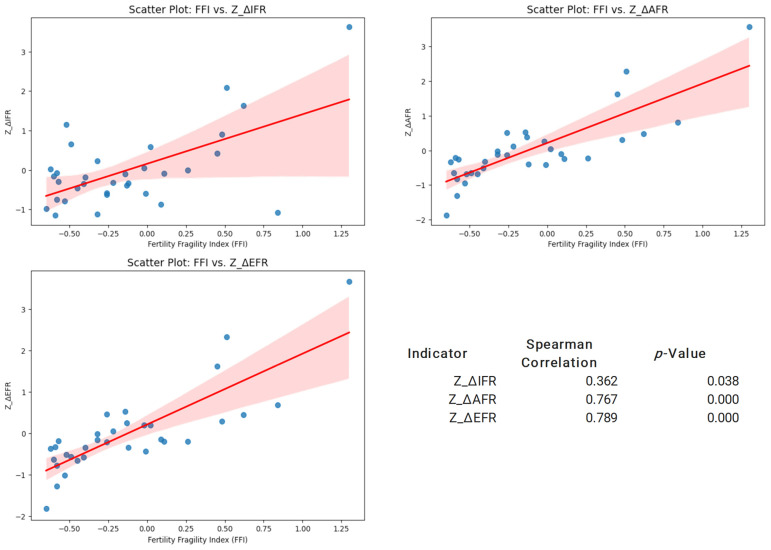
Relationship Between Early Fertility Fragility Index (EFFI) and Changes in Fertility Indicators. This matrix presents scatter plots with trend lines showing the association between EFFI and standardized changes in fertility indicators (ΔIFR, ΔAFR, and ΔEFR). The bottom-right panel summarizes Spearman correlation coefficients and *p*-values, highlighting the statistical significance of these relationships.

The Spearman correlation analysis indicates a positive association between the Early Fertility Fragility Index (EFFI) and changes in fertility rates. The correlation between EFFI and Z_ΔIFR is 0.33 (*p* = 0.061), suggesting a weak but not statistically significant relationship. In contrast, the correlations with Z_ΔAFR (0.736, *p* < 0.001) and Z_ΔEFR (0.762, *p* < 0.001) are strong and statistically significant, confirming that higher fragility scores are associated with greater increases in early fertility rates (See Figure 5).

## 4. Discussion

This study explores the relationship between early fertility, domestic violence, and structural inequalities in Colombia’s departments, particularly in the post-pandemic period. By employing a Generalized Linear Model (GLM), we refined the definition of the Early Fertility Fragility Index (EFFI) and assessed its association with key risk factors. This methodological approach provides a quantitative framework for understanding how structural vulnerabilities contribute to early fertility rates, offering valuable insights for policy interventions [16].

The results indicate significant regional disparities in EFFI, with the highest values observed in Vaupés, Arauca, and La Guajira, suggesting that these regions experience heightened social fragility and associated risks [14]. Conversely, Bogotá D.C., Risaralda, and Santander exhibited the lowest EFFI values, highlighting the protective effects of more stable social and economic infrastructures.. The GLM analysis confirms that certain risk indicators—including the percentage of Indigenous and migrant-affiliated women, domestic violence rates, child homicide rates, and suicide rates were strongly associated with higher EFFI scores. These findings align with previous research emphasizing the role of structural inequalities in shaping early fertility outcomes [3,13,17], as well as international studies highlighting how socioeconomic disparities exacerbate vulnerabilities during periods of crisis [6,7,11,13,18]. Additionally, the Spearman correlation analysis validates the robustness of the EFFI, reinforcing its potential as a reliable tool for assessing regional disparities.

Despite growing policy efforts to address early fertility, current strategies in Colombia lack the necessary territorial focus to tailor interventions based on structural vulnerabilities and regional needs. The existing frameworks, such as the ICBF manual and Profamilia’s programs represent valuable steps but require further adaptation to incorporate risk segmentation and resilience-building approaches [18,19].

Implications for public policies: The findings underscore the need for regionally differentiated policies that prioritize: Investment in social infrastructure to reduce systemic vulnerabilities; Expanded access to reproductive health services, particularly in underserved areas; Multisectoral strategies that address structural inequalities and enhance regional resilience; andData-driven resource allocation, using the EFFI as a tool to target interventions effectively.

Theoretical Considerations and Methodological Assumptions: This study was well-grounded in existing literature on social fragility and early fertility. The EFFI integrates multiple dimensions of vulnerability, but its construction relies on specific assumptions, such as maintaining an equal weight for the different indicator; future research should explore alternative weighting methods, such as principal component analysis (PCA) or machine learning techniques, to assess whether different configurations yield similar predictive power [7,19].

Limitations and opportunities for future research: This study is subject to certain limitations, primarily related to the use of secondary data; potential underreporting of violence and birth indicators, which may affect the accuracy of EFFI scores; the GLM approach assumes linear relationships, which may not fully capture the complex interactions between structural risk factors.

To enhance the robustness and applicability of the EFFI, future research should: Explore alternative modeling techniques, such as Bayesian inference or longitudinal analyses, to capture how these risks evolve over time; incorporate qualitative data, such as community-level interviews, to gain deeper insights into sociocultural mechanisms influencing early fertility trends; and validate the EFFI in post-pandemic years, assessing its long-term predictive capacity.

## 5. Conclusions

This study underscores the heterogeneity of early fertility, domestic violence, and social fragility across Colombia, highlighting the critical role of structural inequalities in exacerbating vulnerabilities, particularly during crises such as the COVID-19 pandemic. Regions with a high proportion of Indigenous populations, socioeconomic marginalization, and reliance on the subsidized healthcare system exhibited the highest levels of social fragility, reinforcing long-standing disparities in access to essential services. These structural inequalities directly contribute to increased early fertility rates, emphasizing the need for targeted, evidence-based interventions in the most affected areas.

The Early Fertility Fragility Index (EFFI), a novel metric developed in this study, provides a comprehensive tool for quantifying and comparing fragility levels across regions. By refining the EFFI through a Generalized Linear Model (GLM), we validated its predictive capacity, confirming key risk factors associated with early fertility outcomes. This methodological approach strengthens the framework for data-driven policymaking, allowing for the prioritization of high-risk regions and the design of targeted interventions.

While these findings align with global research on social fragility and early fertility, the EFFI offers a distinct contribution by integrating regional disparities into a quantitative metric, enabling comparative assessments and long-term monitoring. Future research should focus on evaluating post-pandemic fertility trends, refining intervention strategies, and exploring alternative modeling approaches, such as Bayesian inference or longitudinal analyses, to capture the evolving nature of social fragility. Additionally, incorporating qualitative insights will be key to understanding the sociocultural dimensions of early fertility and domestic violence.

By addressing structural determinants of vulnerability and enhancing regional resilience, policymakers can mitigate early fertility risks and promote social equity. These efforts will be essential in reducing disparities, strengthening reproductive health policies, and fostering long-term stability in Colombia’s most affected communities.

## Figures and Tables

**Table 1:** Structural Inequality Indicators by Department (Colombia period 2019 to 2022).

Department	%PIW Median (Min; Max)	%PMW Median (Min; Max)	%PSW Median (Min; Max)
Amazonas	46.08 (45.98; 47.02)	0.02 (0; 0.03)	76.5 (75.65; 76.71)
Antioquia	0.55 (0.53; 0.56)	0.16 (0.01; 0.3)	38.09 (36.18; 39.18)
Arauca	2.92 (2.88; 2.99)	1.07 (0.04; 1.27)	81.16 (80.12; 81.85)
Atlantico	0.27 (0.21; 0.28)	0.21 (0.09; 0.28)	55.42 (54.04; 56.43)
Bogota D.C.	0.08 (0.08; 0.1)	0.4 (0.01; 0.56)	19.34 (16.01; 21.52)
Bolivar	0.03 (0.03; 0.04)	0.31 (0.06; 0.57)	69.32 (67.94; 70.37)
Boyaca	0.48 (0.47; 0.48)	0.06 (0; 0.1)	57.28 (57.14; 58.31)
Caldas	4.64 (4.48; 4.79)	0.07 (0.01; 0.18)	45.48 (44.25; 46.15)
Caqueta	0.92 (0.82; 1.21)	0.01 (0; 0.02)	80.1 (78.92; 80.42)
Casanare	1.47 (1.4; 1.53)	0.14 (0; 0.23)	60.1 (57; 60.42)
Cauca	21.84 (21.33; 21.96)	0.03 (0; 0.16)	76.66 (75.93; 76.91)
Cesar	5.78 (5.38; 5.88)	0.32 (0.01; 0.61)	72.72 (71.18; 73.32)
Choco	10.59 (8.86; 11.87)	0.01 (0; 0.02)	86.07 (85.27; 86.43)
Cordoba	4.9 (4.59; 5.25)	0.02 (0.01; 0.04)	79.03 (78.14; 79.85)
Cundinamarca	0.03 (0.03; 0.06)	0.19 (0; 0.39)	36.51 (35.46; 38.46)
Guainia	60.94 (52.53; 69.51)	0.05 (0.01; 0.44)	87.47 (87.05; 88.68)
Guaviare	7.88 (7.61; 8.7)	0.01 (0; 0.15)	75.69 (75.42; 76)
Huila	0.93 (0.9; 0.95)	0.02 (0; 0.06)	69.88 (68.84; 70.19)
La Guajira	43.31 (41.42; 44.67)	0.58 (0; 0.98)	84.14 (82.65; 84.31)
Magdalena	1.33 (1.26; 1.37)	0.15 (0; 0.5)	70.74 (70.02; 72.04)
Meta	2.18 (2.07; 2.36)	0.04 (0; 0.31)	55.4 (53.73; 56.82)
Narino	11.99 (11.44; 12.41)	0.06 (0.01; 0.32)	79.37 (79.18; 80.03)
Norte de Santander	0.17 (0.15; 0.18)	0.5 (0; 2.43)	70.76 (69.18; 71.31)
Putumayo	16.56 (16.21; 16.81)	0.1 (0.01; 0.68)	82.96 (82.78; 83.18)
Quindio	0.17 (0.15; 0.22)	0.12 (0.01; 0.15)	47.13 (46.11; 48.16)
Risaralda	2.54 (2.51; 2.61)	0.07 (0; 0.16)	42.74 (40.99; 44.3)
San Andres	0 (0; 0.01)	0.01 (0; 0.01)	29.77 (22.86; 32.58)
Santander	0.06 (0.05; 0.06)	0.12 (0.03; 0.24)	47.03 (44.58; 47.82)
Sucre	10.09 (8.74; 11.11)	0.04 (0; 0.16)	81.5 (81.3; 82.33)
Tolima	3.14 (3.05; 3.27)	0.04 (0; 0.09)	59.55 (58.63; 60.28)
Valle	0.44 (0.43; 0.45)	0.14 (0.02; 0.33)	43.24 (40.92; 44.75)
Vaupes	74.43 (73.46; 76.56)	0 (0; 0)	84.06 (83.73; 84.85)
Vichada	53.67 (51.05; 58.09)	0.06 (0; 0.65)	86.45 (85.34; 87.28)

This table presents the median, minimum, and maximum values of structural inequality indicators across departments in Colombia. Higher values of %PIW (Percentage of Indigenous Affiliated Women), %PMW (Percentage of Migrant Affiliated Women), and %PSW (Percentage of Subsidized Affiliated Women) indicate higher levels of indigenous, migrant, and subsidized-affiliated women, respectively, potentially reflecting socio-economic vulnerabilities.

**Table 2:** Exposure to Risk Factors by Department (Colombia period 2019 to 2022).

Department	%DVAW Median (Min; Max)	%DVAC Median (Min; Max)	CHR Median (Min; Max)	SR Median (Min; Max)
Amazonas	291.41 (0; 631.58)	144.32 (122.99; 226.99)	8.12 (5.26; 18.43)	49.4 (36.87; 68.42)
Antioquia	302.03 (0; 621.39)	114.23 (101.18; 128.25)	6.1 (4.6; 10.12)	12.27 (9.66; 13.84)
Arauca	289.96 (0; 586.96)	178.32 (163.97; 194.19)	14.08 (8.22; 24.5)	20.59 (17.29; 21.3)
Atlantico	340.42 (0; 699.46)	75.27 (60.67; 83.12)	3.73 (3.04; 4.6)	5.01 (4.24; 5.38)
Bogota D.C.	289.66 (0; 607.85)	128.97 (115.7; 185.43)	1.87 (1.43; 3.3)	9.07 (6.97; 11.15)
Bolivar	356.58 (0; 735.33)	70.1 (62.9; 82.85)	3.66 (2.92; 4.71)	6.74 (5.19; 8.75)
Boyaca	311.26 (0; 636.31)	107.95 (95.73; 127.71)	1.27 (0.45; 1.58)	11.84 (10.29; 12.72)
Caldas	299.89 (0; 673.47)	104.32 (43.69; 202.73)	2.26 (1.66; 2.65)	11.56 (10.51; 14.87)
Caqueta	360.23 (0; 827.81)	66.32 (59.6; 109.09)	8 (3.94; 19.07)	10.91 (9.09; 12.92)
Casanare	305.25 (0; 635.79)	149.14 (107.96; 181.08)	5.41 (2.93; 6.02)	17.39 (13.65; 20.64)
Cauca	350.44 (0; 724.06)	58.17 (46.57; 81.68)	14.18 (12.1; 15.06)	12.57 (11.94; 17.75)
Cesar	345.29 (0; 721.37)	80.59 (59.89; 111.11)	4.33 (3.26; 7.44)	11.53 (8.4; 15.04)
Choco	403.27 (0; 821.43)	57.71 (53.44; 74.4)	24.76 (21.49; 33.37)	13.28 (8.15; 18.18)
Cordoba	369.83 (0; 811.99)	67.46 (59.95; 94.97)	2.72 (1.79; 5.27)	7.23 (6.12; 8.35)
Cundinamarca	307.32 (0; 645.17)	109.93 (107.16; 151.38)	2.64 (1.46; 3.78)	12.46 (9.63; 15.47)
Guainia	312.5 (0; 686.05)	147.92 (89.55; 174.42)	3.47 (0; 11.49)	18.62 (13.89; 34.78)
Guaviare	257.81 (0; 777.78)	115.83 (88.89; 343.75)	6.07 (3.86; 12.45)	18.1 (9.88; 35.97)
Huila	338.43 (0; 718.83)	66.69 (53.58; 84.41)	4.95 (3; 5.85)	12.89 (12.27; 13.48)
La Guajira	320.59 (0; 745.36)	99.3 (82.23; 146.65)	6.08 (2.75; 7.99)	8.99 (6.43; 11.41)
Magdalena	342.25 (0; 724.42)	72.72 (60.32; 92.4)	4.84 (3.6; 6.22)	8.42 (4.53; 9.42)
Meta	328.86 (0; 667.22)	136.15 (115.89; 161.63)	4.76 (3.16; 7.74)	10.72 (7.53; 15.04)
Narino	352.96 (0; 730.26)	47.65 (42.65; 58.11)	5.94 (4.74; 9.06)	13.29 (11.56; 16.97)
Norte de Santander	328 (0; 706.23)	89.99 (73.41; 108)	3.8 (3.54; 4.34)	9.2 (7.92; 12.54)
Putumayo	345.09 (0; 712.03)	100.77 (60.85; 134.62)	11.1 (6.53; 13.43)	16.76 (11.1; 23.2)
Quindio	309.41 (0; 681.59)	104.93 (84.58; 114.38)	3.83 (3.17; 7.6)	9.79 (8.45; 11.9)
Risaralda	308.27 (0; 704)	97.09 (88.91; 183.92)	2.47 (2.2; 3.49)	10.21 (9.8; 13.65)
San Andres	311.19 (0; 653.47)	88.97 (70.51; 111.89)	17.74 (8.37; 30.47)	10.96 (0; 13.94)
Santander	315.23 (0; 642.6)	102.83 (92.42; 114.38)	2.66 (1.7; 3.53)	9.05 (8.43; 13.54)
Sucre	376.09 (0; 818.18)	59.86 (57.58; 75.7)	2.81 (2.11; 3.97)	9.86 (9.01; 11.1)
Tolima	319.58 (0; 668.6)	97.73 (81.97; 144.27)	2.84 (2.13; 3.87)	12.51 (10.42; 16.19)
Valle	343.65 (0; 735.57)	67.24 (59.82; 89.37)	8.24 (7.76; 11.64)	7.42 (5.63; 8.33)
Vaupes	263.16 (0; 750)	91.46 (62.5; 105.26)	0 (0; 22.99)	117.95 (103.45; 153.06)
Vichada	0 (0; 0)	735.29 (230.77; 1000)	2.02 (0; 15.96)	11.2 (9.62; 21.28)

This table shows the distribution of domestic violence against women and children, along with child homicide rates. These indicators provide insight into risk factors associated with early fertility. Departments with higher %DVAW (Percentage of Domestic violence against women), %DVAC (Percentage of Domestic Violence Against Children), CHR (Child Homicide Rate per 1000 deaths), and SR (Suicide Rate per 1000 deaths).

**Table 3:** Fertility Indicator by Department (Colombia period 2019 to 2022).

Department	IFR Median (Min; Max)	AFR Median (Min; Max)	EFR Median (Min; Max)
Amazonas	1.96 (1.14; 2.38)	34.81 (33.36; 37.2)	17.93 (17.24; 19.8)
Antioquia	1.4 (1.13; 1.54)	24.85 (20.6; 25.91)	13.8 (11.57; 14.16)
Arauca	2.01 (1.72; 2.33)	34.39 (28.7; 40.22)	18.42 (15.32; 21.04)
Atlantico	1.01 (0.8; 1.17)	34.02 (26.48; 38.68)	17.74 (13.73; 20.36)
Bogota D.C.	0.34 (0.28; 0.4)	14.82 (10.91; 18)	7.63 (5.73; 9.37)
Bolivar	1.74 (1.55; 1.87)	36.93 (33.41; 38.84)	19.46 (17.45; 20.38)
Boyaca	0.62 (0.56; 0.73)	20.53 (17.09; 21.67)	11 (9.19; 11.49)
Caldas	0.86 (0.79; 0.96)	21.33 (19.31; 21.6)	11.85 (10.73; 12)
Caqueta	2.46 (2.16; 2.88)	37.55 (31.3; 43.02)	20.35 (17.09; 23.14)
Casanare	1.22 (0.97; 1.51)	29.13 (25.26; 30.45)	15.3 (13.2; 15.69)
Cauca	1.69 (1.6; 2.04)	32.5 (27.91; 32.9)	17.41 (15.05; 17.67)
Cesar	1.76 (1.69; 2.12)	42.92 (37.35; 44.41)	22.26 (19.29; 23.12)
Choco	2.53 (1.98; 3.18)	34.2 (26.99; 42.63)	18 (14.08; 22.51)
Cordoba	1.75 (1.43; 1.86)	33.77 (30.91; 34.64)	18.04 (16.71; 18.3)
Cundinamarca	0.7 (0.6; 0.75)	24.94 (19.75; 29.49)	12.97 (10.4; 15.37)
Guainia	4.64 (2.86; 5.48)	51.51 (46.7; 63.42)	26.76 (24.82; 33.37)
Guaviare	2.85 (1.91; 3.05)	35.65 (28.22; 37.38)	19.42 (15.58; 20.57)
Huila	1.46 (1.23; 1.7)	33.23 (29.39; 34.65)	17.86 (15.96; 18.58)
La Guajira	2.1 (1.64; 2.89)	49.21 (43.24; 52.03)	25.08 (21.48; 26.5)
Magdalena	1.85 (1.65; 1.95)	44.15 (38.38; 45.92)	23.21 (20.11; 24.17)
Meta	1.4 (1.01; 1.62)	29.97 (26.35; 31.98)	15.96 (14.19; 16.72)
Narino	1.45 (1.29; 1.67)	23.82 (18.42; 26.31)	13.36 (10.48; 14.35)
Norte de Santander	1.22 (1.12; 1.36)	32.68 (28.62; 35.28)	17.22 (15.26; 18.54)
Putumayo	2.31 (1.66; 2.98)	30.69 (29.43; 32.3)	17.16 (16.32; 18.51)
Quindio	0.9 (0.79; 0.97)	22.46 (18.5; 24.48)	12.58 (10.4; 13.66)
Risaralda	0.96 (0.83; 1.05)	24.91 (19.64; 25.4)	13.61 (10.73; 13.76)
San Andres	0.24 (0.23; 0.69)	21.21 (16.73; 26.52)	10.63 (8.59; 12.94)
Santander	0.71 (0.63; 0.77)	23.36 (19.51; 25.23)	12.19 (10.24; 13.17)
Sucre	1.47 (1.39; 1.59)	34.9 (32.12; 38.1)	18.51 (17.01; 19.92)
Tolima	1.24 (1.11; 1.59)	28.9 (24.34; 30.45)	15.63 (13.26; 16.29)
Valle	0.88 (0.79; 1.06)	21.17 (15.93; 22.67)	11.43 (8.7; 12.27)
Vaupes	2 (1.09; 3.12)	41.14 (38.14; 56.65)	20.99 (18.97; 28.89)
Vichada	5.07 (3.77; 7.33)	70.63 (44.07; 72.21)	36.5 (25.91; 38.24)

The fertility indicators table highlights variations in IFR (Infant Fertility Rate), AFR (Adolescent Fertility Rate), and EFR (Early Fertility Rate) across departments. The interquartile range helps identify departments with higher fertility trends, which may correlate with structural inequalities and exposure to violence.

## Data Availability

The original contributions presented in this study are included in the article/Supplementary Material. Further inquiries can be directed to the corresponding author.

## Supplementary Materials

The following supporting information can be downloaded at: https://www.mdpi.com/article/10.3390/ijerph22030453/s1, Python Notebook: Manuscript-supplementary.html.

## Abbreviations

The following abbreviations are used in this manuscript:

AFR	Adolescent Fertility Rate
CHR	Child Homicide Rate
DOAJ	Directory of Open Access Journals
DVAC	Domestic Violence Against Children
DVAW	Domestic Violence Against Women
EFFI	Early Fertility Fragility Index
EFR	Early Fertility Rate
GLM	Generalized Linear Model
ICBF	Instituto Colombiano de Bienestar Familiar (Colombian Institute of Family Welfare)
IFR	Infant Fertility Rate
LD	Linear Dichroism
OECD	Organisation for Economic Co-operation and Development
PIW	Percentage of Indigenous Affiliated Women
PMW	Percentage of Migrant Affiliated Women
PSW	Percentage of Subsidized Affiliated Women
SR	Suicide Rate
SRH	Sexual and Reproductive Health
TLA	Three-Letter Acronym
UNFPA	United Nations Population Fund
WHO	World Health Organization
