# Supplementary material for: Social Fragility: Impact of Early Fertility and Domestic Violence in Colombia During the Pandemic
Source: Int J Environ Res Public Health. 2025 Mar 20;22(3):453. doi: 10.3390/ijerph22030453 (PMC11942471; doi:10.3390/ijerph22030453)
Supplement: Supplementary file 1 [file ijerph-22-00453-s001.zip › ijerph-3509865-supplementary.html]

EarlyFertilityFragilityIndex (1)


# Carga de datos y calculo de indicadores¶

In [18]:

```
# Fragility Index Diagram (Funnel Layout with Symmetric Distribution)
import matplotlib.pyplot as plt
import networkx as nx

# Data for the diagram (Items → Dimensions → Fragility Index)
indicators = {
    "Structural\nInequality": [
        "Indigenous\nAffiliated Women (%)",
        "Migrant\nAffiliated Women (%)",
        "Subsidized\nAffiliated Women (%)"
    ],
    "Exposure to\nRisk Factors": [
        "Domestic Violence\nAgainst Women (%)",
        "Domestic Violence\nAgainst Children (%)",
        "Suicide Rate",
        "Child\nHomicide Rate"
    ]
}

# Central domain
domain = "Fertility Fragility\nIndex (FFI)"

# Create the graph
G = nx.DiGraph()

# Add nodes and edges
for dimension, items in indicators.items():
    G.add_node(dimension, color='#ADD8E6', size=3200, shape='ellipse', layer=1)  # Middle layer (Dimensions)
    for item in items:
        G.add_node(item, color='white', size=2500, shape='ellipse', layer=0)  # Left layer (Items)
        G.add_edge(item, dimension)

# Add domain as the final node
G.add_node(domain, color='#FFD700', size=5000, shape='ellipse', layer=2)  # Rightmost layer
for dimension in indicators.keys():
    G.add_edge(dimension, domain)

# Define positions (funnel layout with symmetric distribution)
pos = {}
x_spacing = 3  # Horizontal spacing
y_max = 5  # Maximum height for symmetry
y_min = -5  # Minimum height for symmetry
y_step = (y_max - y_min) / (len(G.nodes) // 3)  # Dynamic step for symmetry

# Assign positions for Items (leftmost layer)
y_position = y_max
for dimension, items in indicators.items():
    for i, item in enumerate(items):
        pos[item] = (-x_spacing, y_position)
        y_position -= y_step  # Uniform spacing

# Assign positions for Dimensions (middle layer)
y_position = (y_max + y_min) / 2  # Centered start
x_pos = 0
for dimension in indicators.keys():
    pos[dimension] = (x_pos, y_position)
    y_position -= y_step * 2  # Balanced spacing

# Assign position for the Domain (rightmost, centered)
pos[domain] = (x_spacing * 2, 0)  # True centered position

# Draw the graph
plt.figure(figsize=(14, 8))

# Draw edges with gray dashed lines
nx.draw_networkx_edges(G, pos, edge_color='gray', style="dashed", arrows=True, arrowstyle='-|>', arrowsize=20)

# Draw nodes with ellipses
for node in G.nodes:
    node_size = nx.get_node_attributes(G, 'size')[node]
    node_color = nx.get_node_attributes(G, 'color')[node]
    nx.draw_networkx_nodes(G, pos, nodelist=[node], node_color=node_color, node_size=node_size, node_shape='o')

# Draw labels
nx.draw_networkx_labels(G, pos, font_size=10, font_weight="bold")

plt.title("Fertility Fragility Index (FFI): Symmetric Funnel-Shaped Map", fontsize=14, fontweight="bold")
plt.tight_layout()
plt.show()
```

# Descriptive table and HeatMap¶

In [23]:

```
# Install required libraries (if not already installed)
!pip install pandas numpy --quiet

import pandas as pd
import numpy as np

# ==== 1. LOAD DATA ====
file_path = '/content/drive/MyDrive/Sintesis Evidencia/Tadeo/MiTesis/Articulos/Articulo1/Datos/LibroIntegrado.xlsx'
sheet_name = 'Integrado'
data = pd.read_excel(file_path, sheet_name=sheet_name)

# Filter data for 'Year' <= 2022
data = data[data['Año'] <= 2022]

# Pivot data for period indicators
pivot_period_data = data.pivot_table(
    index=['Departamento', 'Año'],  # Include 'Año' for period calculations
    columns='Attribute',
    values='Value',
    aggfunc='sum'
).reset_index()

# ==== 2. CALCULATE INDICATORS ====
# Structural Inequality Indicators
pivot_period_data['%PIW'] = (pivot_period_data['Afiliadas Indigenas'] / pivot_period_data['Total Afiliadas']) * 100
pivot_period_data['%PMW'] = (pivot_period_data['Afiliadas Migrantes'] / pivot_period_data['Total Afiliadas']) * 100
pivot_period_data['%PSW'] = (pivot_period_data['Afiliadas R_Subsidiado'] / pivot_period_data['Total Afiliadas']) * 100

# Exposure to Risk Factors
pivot_period_data['%DVAW'] = (pivot_period_data['Violencia_intrafamiliar.VI_contra la mujer'] /
                              pivot_period_data['Violencia_intrafamiliar.Casos de violencia intrafamiliar (VI)']) * 100
pivot_period_data['%DVAC'] = (pivot_period_data['Violencia_intrafamiliar.VI_contra Infancia y adolescencia'] /
                              pivot_period_data['Violencia_intrafamiliar.Casos de violencia intrafamiliar (VI)']) * 100
pivot_period_data['CHR'] = (pivot_period_data['Homicidio a menor de edad'] / pivot_period_data['Total defunciones']) * 1000
pivot_period_data['SR'] = (pivot_period_data['Suicidio'] / pivot_period_data['Total defunciones']) * 1000


# Fertility Indicators
pivot_period_data['IFR'] = (pivot_period_data['Fertilidad Infantil (10 a 14 años)'] / pivot_period_data['Afiliadas 10 a 14 años']) * 1000
pivot_period_data['AFR'] = (pivot_period_data['Fertilidad Adolescente (15 a 19 años)'] / pivot_period_data['Afiliadas 15 a 19 años']) * 1000
pivot_period_data['EFR'] = ((pivot_period_data['Fertilidad Infantil (10 a 14 años)'] +
                             pivot_period_data['Fertilidad Adolescente (15 a 19 años)']) / pivot_period_data['Afiliadas 10 a 19 años']) * 1000

# Rename "Departamento" to "Department"
pivot_period_data.rename(columns={'Departamento': 'Department'}, inplace=True)

# Select relevant columns
columns_to_keep = ['Department', 'Año', '%PIW', '%PMW', '%PSW', '%DVAW', '%DVAC', 'CHR','SR', 'IFR', 'AFR', 'EFR']
final_data = pivot_period_data[columns_to_keep]

# ==== 3. COMPUTE MEDIANS AND INTERQUARTILE RANGES ====
def compute_summary(df, indicators):
    summary = df.groupby("Department")[indicators].agg([
        ("Median", np.median),
        ("Min", lambda x: np.min(x)),
        ("Max", lambda x: np.max(x))
    ])
    summary = summary.round(2)
    return summary

# Compute tables for different indicator groups
table1 = compute_summary(final_data, ['%PIW', '%PMW', '%PSW'])
table2 = compute_summary(final_data, ['%DVAW', '%DVAC', 'CHR', 'SR'])
table3 = compute_summary(final_data, ['IFR', 'AFR', 'EFR'])

# ==== 4. DISPLAY TABLES WITH DESCRIPTIONS ====
print("\n **Table 1: Structural Inequality Indicators**")
display(table1)
print("\nThis table presents the median, minimum, and maximum values of structural inequality indicators across departments in Colombia. Higher values of %PIW (Percentage of Indigenous Affiliated Women), %PMW (Percentage of Migrant Affiliated Women), and %PSW (Percentage of Subsidized Affiliated Women) indicate higher levels of indigenous, migrant, and subsidized-affiliated women, respectively, potentially reflecting socio-economic vulnerabilities.")

print("\n**Table 2: Exposure to Risk Factors**")
display(table2)
print("\nThis table shows the distribution of domestic violence against women and children, along with child homicide rates. These indicators provide insight into risk factors associated with early fertility. Departments with higher %DVAW (Percentage of Domestic violence against women ), %DVAC (Percentage of Domestic Violence Against Children), CHR (Child Homicide Rate per 1,000), and SR (Suicide Rate per 1,000) may exhibit increased vulnerability to social and health-related disparities.")

print("\n**Table 3: Fertility Indicators**")
display(table3)
print("\nThe fertility indicators table highlights variations in IFR (Infant Fertility Rate), AFR (Adolescent Fertility Rate), and EFR (Early Fertility Rate) across departments. The interquartile range helps identify departments with higher fertility trends, which may correlate with structural inequalities and exposure to violence.")
```

```
 **Table 1: Structural Inequality Indicators**
```

```
<ipython-input-23-174b6bc9f0fc>:53: FutureWarning: The provided callable <function median at 0x7d96f7956200> is currently using SeriesGroupBy.median. In a future version of pandas, the provided callable will be used directly. To keep current behavior pass the string "median" instead.
  summary = df.groupby("Department")[indicators].agg([
<ipython-input-23-174b6bc9f0fc>:53: FutureWarning: The provided callable <function median at 0x7d96f7956200> is currently using SeriesGroupBy.median. In a future version of pandas, the provided callable will be used directly. To keep current behavior pass the string "median" instead.
  summary = df.groupby("Department")[indicators].agg([
<ipython-input-23-174b6bc9f0fc>:53: FutureWarning: The provided callable <function median at 0x7d96f7956200> is currently using SeriesGroupBy.median. In a future version of pandas, the provided callable will be used directly. To keep current behavior pass the string "median" instead.
  summary = df.groupby("Department")[indicators].agg([
<ipython-input-23-174b6bc9f0fc>:53: FutureWarning: The provided callable <function median at 0x7d96f7956200> is currently using SeriesGroupBy.median. In a future version of pandas, the provided callable will be used directly. To keep current behavior pass the string "median" instead.
  summary = df.groupby("Department")[indicators].agg([
<ipython-input-23-174b6bc9f0fc>:53: FutureWarning: The provided callable <function median at 0x7d96f7956200> is currently using SeriesGroupBy.median. In a future version of pandas, the provided callable will be used directly. To keep current behavior pass the string "median" instead.
  summary = df.groupby("Department")[indicators].agg([
<ipython-input-23-174b6bc9f0fc>:53: FutureWarning: The provided callable <function median at 0x7d96f7956200> is currently using SeriesGroupBy.median. In a future version of pandas, the provided callable will be used directly. To keep current behavior pass the string "median" instead.
  summary = df.groupby("Department")[indicators].agg([
```

| Attribute | %PIW | | | %PMW | | | %PSW | | |
| --- | --- | --- | --- | --- | --- | --- | --- | --- | --- |
|  | Median | Min | Max | Median | Min | Max | Median | Min | Max |
| Department |  |  |  |  |  |  |  |  |  |
| AMAZONAS | 46.08 | 45.98 | 47.02 | 0.02 | 0.00 | 0.03 | 76.50 | 75.65 | 76.71 |
| ANTIOQUIA | 0.55 | 0.53 | 0.56 | 0.16 | 0.01 | 0.30 | 38.09 | 36.18 | 39.18 |
| ARAUCA | 2.92 | 2.88 | 2.99 | 1.07 | 0.04 | 1.27 | 81.16 | 80.12 | 81.85 |
| ATLANTICO | 0.27 | 0.21 | 0.28 | 0.21 | 0.09 | 0.28 | 55.42 | 54.04 | 56.43 |
| BOGOTA D.C. | 0.08 | 0.08 | 0.10 | 0.40 | 0.01 | 0.56 | 19.34 | 16.01 | 21.52 |
| BOLIVAR | 0.03 | 0.03 | 0.04 | 0.31 | 0.06 | 0.57 | 69.32 | 67.94 | 70.37 |
| BOYACA | 0.48 | 0.47 | 0.48 | 0.06 | 0.00 | 0.10 | 57.28 | 57.14 | 58.31 |
| CALDAS | 4.64 | 4.48 | 4.79 | 0.07 | 0.01 | 0.18 | 45.48 | 44.25 | 46.15 |
| CAQUETA | 0.92 | 0.82 | 1.21 | 0.01 | 0.00 | 0.02 | 80.10 | 78.92 | 80.42 |
| CASANARE | 1.47 | 1.40 | 1.53 | 0.14 | 0.00 | 0.23 | 60.10 | 57.00 | 60.42 |
| CAUCA | 21.84 | 21.33 | 21.96 | 0.03 | 0.00 | 0.16 | 76.66 | 75.93 | 76.91 |
| CESAR | 5.78 | 5.38 | 5.88 | 0.32 | 0.01 | 0.61 | 72.72 | 71.18 | 73.32 |
| CHOCO | 10.59 | 8.86 | 11.87 | 0.01 | 0.00 | 0.02 | 86.07 | 85.27 | 86.43 |
| CORDOBA | 4.90 | 4.59 | 5.25 | 0.02 | 0.01 | 0.04 | 79.03 | 78.14 | 79.85 |
| CUNDINAMARCA | 0.03 | 0.03 | 0.06 | 0.19 | 0.00 | 0.39 | 36.51 | 35.46 | 38.46 |
| GUAINIA | 60.94 | 52.53 | 69.51 | 0.05 | 0.01 | 0.44 | 87.47 | 87.05 | 88.68 |
| GUAVIARE | 7.88 | 7.61 | 8.70 | 0.01 | 0.00 | 0.15 | 75.69 | 75.42 | 76.00 |
| HUILA | 0.93 | 0.90 | 0.95 | 0.02 | 0.00 | 0.06 | 69.88 | 68.84 | 70.19 |
| LA GUAJIRA | 43.31 | 41.42 | 44.67 | 0.58 | 0.00 | 0.98 | 84.14 | 82.65 | 84.31 |
| MAGDALENA | 1.33 | 1.26 | 1.37 | 0.15 | 0.00 | 0.50 | 70.74 | 70.02 | 72.04 |
| META | 2.18 | 2.07 | 2.36 | 0.04 | 0.00 | 0.31 | 55.40 | 53.73 | 56.82 |
| NARINO | 11.99 | 11.44 | 12.41 | 0.06 | 0.01 | 0.32 | 79.37 | 79.18 | 80.03 |
| NORTE DE SANTANDER | 0.17 | 0.15 | 0.18 | 0.50 | 0.00 | 2.43 | 70.76 | 69.18 | 71.31 |
| PUTUMAYO | 16.56 | 16.21 | 16.81 | 0.10 | 0.01 | 0.68 | 82.96 | 82.78 | 83.18 |
| QUINDIO | 0.17 | 0.15 | 0.22 | 0.12 | 0.01 | 0.15 | 47.13 | 46.11 | 48.16 |
| RISARALDA | 2.54 | 2.51 | 2.61 | 0.07 | 0.00 | 0.16 | 42.74 | 40.99 | 44.30 |
| SAN ANDRES | 0.00 | 0.00 | 0.01 | 0.01 | 0.00 | 0.01 | 29.77 | 22.86 | 32.58 |
| SANTANDER | 0.06 | 0.05 | 0.06 | 0.12 | 0.03 | 0.24 | 47.03 | 44.58 | 47.82 |
| SUCRE | 10.09 | 8.74 | 11.11 | 0.04 | 0.00 | 0.16 | 81.50 | 81.30 | 82.33 |
| TOLIMA | 3.14 | 3.05 | 3.27 | 0.04 | 0.00 | 0.09 | 59.55 | 58.63 | 60.28 |
| VALLE | 0.44 | 0.43 | 0.45 | 0.14 | 0.02 | 0.33 | 43.24 | 40.92 | 44.75 |
| VAUPES | 74.43 | 73.46 | 76.56 | 0.00 | 0.00 | 0.00 | 84.06 | 83.73 | 84.85 |
| VICHADA | 53.67 | 51.05 | 58.09 | 0.06 | 0.00 | 0.65 | 86.45 | 85.34 | 87.28 |

```
This table presents the median, minimum, and maximum values of structural inequality indicators across departments in Colombia. Higher values of %PIW (Percentage of Indigenous Affiliated Women), %PMW (Percentage of Migrant Affiliated Women), and %PSW (Percentage of Subsidized Affiliated Women) indicate higher levels of indigenous, migrant, and subsidized-affiliated women, respectively, potentially reflecting socio-economic vulnerabilities.

**Table 2: Exposure to Risk Factors**
```

| Attribute | %DVAW | | | %DVAC | | | CHR | | | SR | | |
| --- | --- | --- | --- | --- | --- | --- | --- | --- | --- | --- | --- | --- |
|  | Median | Min | Max | Median | Min | Max | Median | Min | Max | Median | Min | Max |
| Department |  |  |  |  |  |  |  |  |  |  |  |  |
| AMAZONAS | 29.14 | 0.0 | 63.16 | 14.43 | 12.30 | 22.70 | 8.12 | 5.26 | 18.43 | 49.40 | 36.87 | 68.42 |
| ANTIOQUIA | 30.20 | 0.0 | 62.14 | 11.42 | 10.12 | 12.83 | 6.10 | 4.60 | 10.12 | 12.27 | 9.66 | 13.84 |
| ARAUCA | 29.00 | 0.0 | 58.70 | 17.83 | 16.40 | 19.42 | 14.08 | 8.22 | 24.50 | 20.59 | 17.29 | 21.30 |
| ATLANTICO | 34.04 | 0.0 | 69.95 | 7.53 | 6.07 | 8.31 | 3.73 | 3.04 | 4.60 | 5.01 | 4.24 | 5.38 |
| BOGOTA D.C. | 28.97 | 0.0 | 60.79 | 12.90 | 11.57 | 18.54 | 1.87 | 1.43 | 3.30 | 9.07 | 6.97 | 11.15 |
| BOLIVAR | 35.66 | 0.0 | 73.53 | 7.01 | 6.29 | 8.29 | 3.66 | 2.92 | 4.71 | 6.74 | 5.19 | 8.75 |
| BOYACA | 31.13 | 0.0 | 63.63 | 10.79 | 9.57 | 12.77 | 1.27 | 0.45 | 1.58 | 11.84 | 10.29 | 12.72 |
| CALDAS | 29.99 | 0.0 | 67.35 | 10.43 | 4.37 | 20.27 | 2.26 | 1.66 | 2.65 | 11.56 | 10.51 | 14.87 |
| CAQUETA | 36.02 | 0.0 | 82.78 | 6.63 | 5.96 | 10.91 | 8.00 | 3.94 | 19.07 | 10.91 | 9.09 | 12.92 |
| CASANARE | 30.52 | 0.0 | 63.58 | 14.91 | 10.80 | 18.11 | 5.41 | 2.93 | 6.02 | 17.39 | 13.65 | 20.64 |
| CAUCA | 35.04 | 0.0 | 72.41 | 5.82 | 4.66 | 8.17 | 14.18 | 12.10 | 15.06 | 12.57 | 11.94 | 17.75 |
| CESAR | 34.53 | 0.0 | 72.14 | 8.06 | 5.99 | 11.11 | 4.33 | 3.26 | 7.44 | 11.53 | 8.40 | 15.04 |
| CHOCO | 40.33 | 0.0 | 82.14 | 5.77 | 5.34 | 7.44 | 24.76 | 21.49 | 33.37 | 13.28 | 8.15 | 18.18 |
| CORDOBA | 36.98 | 0.0 | 81.20 | 6.75 | 5.99 | 9.50 | 2.72 | 1.79 | 5.27 | 7.23 | 6.12 | 8.35 |
| CUNDINAMARCA | 30.73 | 0.0 | 64.52 | 10.99 | 10.72 | 15.14 | 2.64 | 1.46 | 3.78 | 12.46 | 9.63 | 15.47 |
| GUAINIA | 31.25 | 0.0 | 68.60 | 14.79 | 8.96 | 17.44 | 3.47 | 0.00 | 11.49 | 18.62 | 13.89 | 34.78 |
| GUAVIARE | 25.78 | 0.0 | 77.78 | 11.58 | 8.89 | 34.38 | 6.07 | 3.86 | 12.45 | 18.10 | 9.88 | 35.97 |
| HUILA | 33.84 | 0.0 | 71.88 | 6.67 | 5.36 | 8.44 | 4.95 | 3.00 | 5.85 | 12.89 | 12.27 | 13.48 |
| LA GUAJIRA | 32.06 | 0.0 | 74.54 | 9.93 | 8.22 | 14.66 | 6.08 | 2.75 | 7.99 | 8.99 | 6.43 | 11.41 |
| MAGDALENA | 34.22 | 0.0 | 72.44 | 7.27 | 6.03 | 9.24 | 4.84 | 3.60 | 6.22 | 8.42 | 4.53 | 9.42 |
| META | 32.89 | 0.0 | 66.72 | 13.62 | 11.59 | 16.16 | 4.76 | 3.16 | 7.74 | 10.72 | 7.53 | 15.04 |
| NARINO | 35.30 | 0.0 | 73.03 | 4.76 | 4.26 | 5.81 | 5.94 | 4.74 | 9.06 | 13.29 | 11.56 | 16.97 |
| NORTE DE SANTANDER | 32.80 | 0.0 | 70.62 | 9.00 | 7.34 | 10.80 | 3.80 | 3.54 | 4.34 | 9.20 | 7.92 | 12.54 |
| PUTUMAYO | 34.51 | 0.0 | 71.20 | 10.08 | 6.08 | 13.46 | 11.10 | 6.53 | 13.43 | 16.76 | 11.10 | 23.20 |
| QUINDIO | 30.94 | 0.0 | 68.16 | 10.49 | 8.46 | 11.44 | 3.83 | 3.17 | 7.60 | 9.79 | 8.45 | 11.90 |
| RISARALDA | 30.83 | 0.0 | 70.40 | 9.71 | 8.89 | 18.39 | 2.47 | 2.20 | 3.49 | 10.21 | 9.80 | 13.65 |
| SAN ANDRES | 31.12 | 0.0 | 65.35 | 8.90 | 7.05 | 11.19 | 17.74 | 8.37 | 30.47 | 10.96 | 0.00 | 13.94 |
| SANTANDER | 31.52 | 0.0 | 64.26 | 10.28 | 9.24 | 11.44 | 2.66 | 1.70 | 3.53 | 9.05 | 8.43 | 13.54 |
| SUCRE | 37.61 | 0.0 | 81.82 | 5.99 | 5.76 | 7.57 | 2.81 | 2.11 | 3.97 | 9.86 | 9.01 | 11.10 |
| TOLIMA | 31.96 | 0.0 | 66.86 | 9.77 | 8.20 | 14.43 | 2.84 | 2.13 | 3.87 | 12.51 | 10.42 | 16.19 |
| VALLE | 34.37 | 0.0 | 73.56 | 6.72 | 5.98 | 8.94 | 8.24 | 7.76 | 11.64 | 7.42 | 5.63 | 8.33 |
| VAUPES | 26.32 | 0.0 | 75.00 | 9.15 | 6.25 | 10.53 | 0.00 | 0.00 | 22.99 | 117.95 | 103.45 | 153.06 |
| VICHADA | 0.00 | 0.0 | 0.00 | 73.53 | 23.08 | 100.00 | 2.02 | 0.00 | 15.96 | 11.20 | 9.62 | 21.28 |

```
This table shows the distribution of domestic violence against women and children, along with child homicide rates. These indicators provide insight into risk factors associated with early fertility. Departments with higher %DVAW (Percentage of Domestic violence against women ), %DVAC (Percentage of Domestic Violence Against Children), CHR (Child Homicide Rate per 1,000), and SR (Suicide Rate per 1,000) may exhibit increased vulnerability to social and health-related disparities.

**Table 3: Fertility Indicators**
```

| Attribute | IFR | | | AFR | | | EFR | | |
| --- | --- | --- | --- | --- | --- | --- | --- | --- | --- |
|  | Median | Min | Max | Median | Min | Max | Median | Min | Max |
| Department |  |  |  |  |  |  |  |  |  |
| AMAZONAS | 1.96 | 1.14 | 2.38 | 34.81 | 33.36 | 37.20 | 17.93 | 17.24 | 19.80 |
| ANTIOQUIA | 1.40 | 1.13 | 1.54 | 24.85 | 20.60 | 25.91 | 13.80 | 11.57 | 14.16 |
| ARAUCA | 2.01 | 1.72 | 2.33 | 34.39 | 28.70 | 40.22 | 18.42 | 15.32 | 21.04 |
| ATLANTICO | 1.01 | 0.80 | 1.17 | 34.02 | 26.48 | 38.68 | 17.74 | 13.73 | 20.36 |
| BOGOTA D.C. | 0.34 | 0.28 | 0.40 | 14.82 | 10.91 | 18.00 | 7.63 | 5.73 | 9.37 |
| BOLIVAR | 1.74 | 1.55 | 1.87 | 36.93 | 33.41 | 38.84 | 19.46 | 17.45 | 20.38 |
| BOYACA | 0.62 | 0.56 | 0.73 | 20.53 | 17.09 | 21.67 | 11.00 | 9.19 | 11.49 |
| CALDAS | 0.86 | 0.79 | 0.96 | 21.33 | 19.31 | 21.60 | 11.85 | 10.73 | 12.00 |
| CAQUETA | 2.46 | 2.16 | 2.88 | 37.55 | 31.30 | 43.02 | 20.35 | 17.09 | 23.14 |
| CASANARE | 1.22 | 0.97 | 1.51 | 29.13 | 25.26 | 30.45 | 15.30 | 13.20 | 15.69 |
| CAUCA | 1.69 | 1.60 | 2.04 | 32.50 | 27.91 | 32.90 | 17.41 | 15.05 | 17.67 |
| CESAR | 1.76 | 1.69 | 2.12 | 42.92 | 37.35 | 44.41 | 22.26 | 19.29 | 23.12 |
| CHOCO | 2.53 | 1.98 | 3.18 | 34.20 | 26.99 | 42.63 | 18.00 | 14.08 | 22.51 |
| CORDOBA | 1.75 | 1.43 | 1.86 | 33.77 | 30.91 | 34.64 | 18.04 | 16.71 | 18.30 |
| CUNDINAMARCA | 0.70 | 0.60 | 0.75 | 24.94 | 19.75 | 29.49 | 12.97 | 10.40 | 15.37 |
| GUAINIA | 4.64 | 2.86 | 5.48 | 51.51 | 46.70 | 63.42 | 26.76 | 24.82 | 33.37 |
| GUAVIARE | 2.85 | 1.91 | 3.05 | 35.65 | 28.22 | 37.38 | 19.42 | 15.58 | 20.57 |
| HUILA | 1.46 | 1.23 | 1.70 | 33.23 | 29.39 | 34.65 | 17.86 | 15.96 | 18.58 |
| LA GUAJIRA | 2.10 | 1.64 | 2.89 | 49.21 | 43.24 | 52.03 | 25.08 | 21.48 | 26.50 |
| MAGDALENA | 1.85 | 1.65 | 1.95 | 44.15 | 38.38 | 45.92 | 23.21 | 20.11 | 24.17 |
| META | 1.40 | 1.01 | 1.62 | 29.97 | 26.35 | 31.98 | 15.96 | 14.19 | 16.72 |
| NARINO | 1.45 | 1.29 | 1.67 | 23.82 | 18.42 | 26.31 | 13.36 | 10.48 | 14.35 |
| NORTE DE SANTANDER | 1.22 | 1.12 | 1.36 | 32.68 | 28.62 | 35.28 | 17.22 | 15.26 | 18.54 |
| PUTUMAYO | 2.31 | 1.66 | 2.98 | 30.69 | 29.43 | 32.30 | 17.16 | 16.32 | 18.51 |
| QUINDIO | 0.90 | 0.79 | 0.97 | 22.46 | 18.50 | 24.48 | 12.58 | 10.40 | 13.66 |
| RISARALDA | 0.96 | 0.83 | 1.05 | 24.91 | 19.64 | 25.40 | 13.61 | 10.73 | 13.76 |
| SAN ANDRES | 0.24 | 0.23 | 0.69 | 21.21 | 16.73 | 26.52 | 10.63 | 8.59 | 12.94 |
| SANTANDER | 0.71 | 0.63 | 0.77 | 23.36 | 19.51 | 25.23 | 12.19 | 10.24 | 13.17 |
| SUCRE | 1.47 | 1.39 | 1.59 | 34.90 | 32.12 | 38.10 | 18.51 | 17.01 | 19.92 |
| TOLIMA | 1.24 | 1.11 | 1.59 | 28.90 | 24.34 | 30.45 | 15.63 | 13.26 | 16.29 |
| VALLE | 0.88 | 0.79 | 1.06 | 21.17 | 15.93 | 22.67 | 11.43 | 8.70 | 12.27 |
| VAUPES | 2.00 | 1.09 | 3.12 | 41.14 | 38.14 | 56.65 | 20.99 | 18.97 | 28.89 |
| VICHADA | 5.07 | 3.77 | 7.33 | 70.63 | 44.07 | 72.21 | 36.50 | 25.91 | 38.24 |

```
The fertility indicators table highlights variations in IFR (Infant Fertility Rate), AFR (Adolescent Fertility Rate), and EFR (Early Fertility Rate) across departments. The interquartile range helps identify departments with higher fertility trends, which may correlate with structural inequalities and exposure to violence.
```

# EFR heatmap trend¶

In [36]:

```
import pandas as pd
import numpy as np
import matplotlib.pyplot as plt
import seaborn as sns

# ==== 1. LOAD DATA ====
file_path = '/content/drive/MyDrive/Sintesis Evidencia/Tadeo/MiTesis/Articulos/Articulo1/Datos/LibroIntegrado.xlsx'
sheet_name = 'Integrado'
data = pd.read_excel(file_path, sheet_name=sheet_name)

# Filter data for 'Year' <= 2022
data = data[data['Año'] <= 2022]

# Pivot data for period indicators
pivot_period_data = data.pivot_table(
    index=['Departamento', 'Año'],  # Include 'Año' for heatmap
    columns='Attribute',
    values='Value',
    aggfunc='sum'
).reset_index()

# EFR Indicator
pivot_period_data['EFR'] = ((pivot_period_data['Fertilidad Infantil (10 a 14 años)'] + pivot_period_data['Fertilidad Adolescente (15 a 19 años)']) / pivot_period_data['Afiliadas 10 a 19 años']) * 1000

# Rename "Departamento" to "Department"
pivot_period_data.rename(columns={'Departamento': 'Department'}, inplace=True)

# ====  CREATE HEATMAP FOR EFR ====
# Pivot data for heatmap
heatmap_data = final_data.pivot(index="Department", columns="Año", values="EFR")

# Sort departments by EFR in the most recent year (descending order)
heatmap_data = heatmap_data.sort_values(by=heatmap_data.columns[-1], ascending=False)

# Plot heatmap
plt.figure(figsize=(12, 10))
sns.heatmap(heatmap_data, cmap="coolwarm", annot=True, fmt=".2f", linewidths=0.5)

# Customize plot
plt.title("Heatmap of Early Fertility Rate (EFR) by Department and Year", fontsize=14)
plt.xlabel("Year")
plt.ylabel("Department")

# Show heatmap
plt.show()

heatmap_data
```

Out[36]:

| Año | 2019 | 2020 | 2021 | 2022 |
| --- | --- | --- | --- | --- |
| Department |  |  |  |  |
| VICHADA | 25.91 | 36.40 | 36.60 | 38.24 |
| GUAINIA | 24.84 | 24.82 | 33.37 | 28.68 |
| LA GUAJIRA | 25.86 | 24.29 | 26.50 | 21.48 |
| VAUPES | 20.96 | 18.97 | 28.89 | 21.02 |
| MAGDALENA | 24.17 | 23.17 | 23.26 | 20.11 |
| CESAR | 22.67 | 21.84 | 23.12 | 19.29 |
| CHOCO | 14.08 | 18.04 | 22.51 | 17.96 |
| BOLIVAR | 19.80 | 19.12 | 20.38 | 17.45 |
| AMAZONAS | 19.80 | 17.24 | 18.45 | 17.42 |
| CAQUETA | 23.14 | 19.36 | 21.35 | 17.09 |
| SUCRE | 19.92 | 18.15 | 18.86 | 17.01 |
| CORDOBA | 18.30 | 17.79 | 18.30 | 16.71 |
| PUTUMAYO | 16.83 | 17.49 | 18.51 | 16.32 |
| HUILA | 18.58 | 17.98 | 17.73 | 15.96 |
| GUAVIARE | 20.03 | 18.81 | 20.57 | 15.58 |
| ARAUCA | 21.04 | 17.35 | 19.49 | 15.32 |
| NORTE DE SANTANDER | 18.54 | 17.12 | 17.33 | 15.26 |
| CAUCA | 17.24 | 17.59 | 17.67 | 15.05 |
| META | 15.82 | 16.72 | 16.09 | 14.19 |
| ATLANTICO | 20.36 | 18.17 | 17.32 | 13.73 |
| TOLIMA | 16.29 | 15.98 | 15.27 | 13.26 |
| CASANARE | 15.69 | 15.23 | 15.38 | 13.20 |
| ANTIOQUIA | 14.16 | 13.75 | 13.85 | 11.57 |
| RISARALDA | 13.70 | 13.76 | 13.52 | 10.73 |
| CALDAS | 12.00 | 11.92 | 11.77 | 10.73 |
| NARINO | 14.35 | 13.45 | 13.27 | 10.48 |
| QUINDIO | 12.93 | 13.66 | 12.24 | 10.40 |
| CUNDINAMARCA | 15.37 | 13.94 | 12.01 | 10.40 |
| SANTANDER | 13.17 | 12.52 | 11.85 | 10.24 |
| BOYACA | 11.49 | 11.41 | 10.59 | 9.19 |
| VALLE | 12.27 | 11.76 | 11.11 | 8.70 |
| SAN ANDRES | 12.94 | 10.78 | 10.48 | 8.59 |
| BOGOTA D.C. | 9.37 | 8.54 | 6.73 | 5.73 |

# Development of EFII¶

In [40]:

```
import pandas as pd
import numpy as np
import statsmodels.api as sm

# ==== 1. LOAD DATA ====
file_path = '/content/drive/MyDrive/Sintesis Evidencia/Tadeo/MiTesis/Articulos/Articulo1/Datos/LibroIntegrado.xlsx'
sheet_name = 'Integrado'
data = pd.read_excel(file_path, sheet_name=sheet_name)

# Filter data for 'Year' <= 2021
data = data[data['Año'] <= 2021]

# Pivot data for period indicators
pivot_period_data = data.pivot_table(
    index=['Departamento', 'Año'],
    columns='Attribute',
    values='Value',
    aggfunc='sum'
).reset_index()

# ==== 2. CREATE THE ANNUAL-LEVEL DATASET ====
annual_data = pivot_period_data.copy()

# Calculate indicators
annual_data['%PIW'] = (annual_data['Afiliadas Indigenas'] / annual_data['Total Afiliadas']) * 100
annual_data['%PMW'] = (annual_data['Afiliadas Migrantes'] / annual_data['Total Afiliadas']) * 100
annual_data['%PSW'] = (annual_data['Afiliadas R_Subsidiado'] / annual_data['Total Afiliadas']) * 100
annual_data['%DVAW'] = (annual_data['Violencia_intrafamiliar.VI_contra la mujer'] /
                         annual_data['Violencia_intrafamiliar.Casos de violencia intrafamiliar (VI)']) * 100
#annual_data['%DVAC'] = (annual_data['Violencia_intrafamiliar.VI_contra Infancia y adolescencia'] /
#                         annual_data['Violencia_intrafamiliar.Casos de violencia intrafamiliar (VI)']) * 100
annual_data['CHR'] = (annual_data['Homicidio a menor de edad'] / annual_data['Total defunciones']) * 1000
annual_data['SR'] = (annual_data['Suicidio'] / annual_data['Total defunciones']) * 1000

# ==== 3. CREATE THE AGGREGATED DATASET (FFI) ====
aggregated_data = annual_data.groupby("Departamento").mean().reset_index()

# Standardize indicators using Z-score
indicators = [
    "%PIW",
    "%PMW",
    "%PSW",
    "%DVAW",
    #"%DVAC",
    "CHR",
    "SR"
              ]
for col in indicators:
    aggregated_data[f"Z_{col}"] = (aggregated_data[col] - aggregated_data[col].mean()) / aggregated_data[col].std()

# Compute the Early Fertility Fragility Index (EFFI)
aggregated_data["FFI"] = aggregated_data[[f"Z_{col}" for col in indicators]].mean(axis=1)

# ==== 4. MERGE DATASETS ====
final_df = pd.merge(annual_data, aggregated_data[['Departamento', 'FFI']], on='Departamento')

# ==== 5. FIT THE GENERALIZED LINEAR MODEL (GLM) ====
X = final_df[indicators]  # Independent variables
X = sm.add_constant(X)  # Add intercept
Y = final_df["FFI"]  # Dependent variable

# Fit the GLM model
model = sm.GLM(Y, X, family=sm.families.Gaussian()).fit()

# Print summary
print(model.summary())
```

```
                 Generalized Linear Model Regression Results                  
==============================================================================
Dep. Variable:                    FFI   No. Observations:                   99
Model:                            GLM   Df Residuals:                       92
Model Family:                Gaussian   Df Model:                            6
Link Function:               Identity   Scale:                        0.036453
Method:                          IRLS   Log-Likelihood:                 27.086
Date:                Mon, 17 Feb 2025   Deviance:                       3.3536
Time:                        23:53:31   Pearson chi2:                     3.35
No. Iterations:                     3   Pseudo R-squ. (CS):             0.9953
Covariance Type:            nonrobust                                         
==============================================================================
                 coef    std err          z      P>|z|      [0.025      0.975]
------------------------------------------------------------------------------
const         -1.3005      0.081    -16.052      0.000      -1.459      -1.142
%PIW           0.0021      0.001      1.431      0.152      -0.001       0.005
%PMW           0.6939      0.103      6.725      0.000       0.492       0.896
%PSW           0.0123      0.001      9.785      0.000       0.010       0.015
%DVAW          0.0018      0.001      2.890      0.004       0.001       0.003
CHR            0.0262      0.003      7.864      0.000       0.020       0.033
SR             0.0098      0.001      7.918      0.000       0.007       0.012
==============================================================================
```

# EFII refined indicator¶

# Compute Fertility Change Indicators (ΔIFR, ΔAFR, ΔEFR) & FFI, and Display a Choropleth Map¶

In [37]:

```
import pandas as pd
import geopandas as gpd
import matplotlib.pyplot as plt
import matplotlib.colors as mcolors
import seaborn as sns
import numpy as np

# ==== 1. LOAD DATA ====
file_path = '/content/drive/MyDrive/Sintesis Evidencia/Tadeo/MiTesis/Articulos/Articulo1/Datos/LibroIntegrado.xlsx'
sheet_name = 'Integrado'
data = pd.read_excel(file_path, sheet_name=sheet_name)

# Filter data for 'Year' <= 2021
data = data[data['Año'] <= 2021]

# Pivot data for period indicators
pivot_period_data = data.pivot_table(
    index=['Departamento', 'Año'],
    columns='Attribute',
    values='Value',
    aggfunc='sum'
).reset_index()

# ==== 2. RECALCULATE INDICATORS ====#'%DVAC' was not included because of low significance in GLM
pivot_period_data['%PIW'] = (pivot_period_data['Afiliadas Indigenas'] / pivot_period_data['Total Afiliadas']) * 100
pivot_period_data['%PMW'] = (pivot_period_data['Afiliadas Migrantes'] / pivot_period_data['Total Afiliadas']) * 100
pivot_period_data['%PSW'] = (pivot_period_data['Afiliadas R_Subsidiado'] / pivot_period_data['Total Afiliadas']) * 100
pivot_period_data['%DVAW'] = (pivot_period_data['Violencia_intrafamiliar.VI_contra la mujer'] /
                              pivot_period_data['Violencia_intrafamiliar.Casos de violencia intrafamiliar (VI)']) * 100
pivot_period_data['CHR'] = (pivot_period_data['Homicidio a menor de edad'] / pivot_period_data['Total defunciones']) * 1000
pivot_period_data['SR'] = (pivot_period_data['Suicidio'] / pivot_period_data['Total defunciones']) * 1000
pivot_period_data['IFR'] = (pivot_period_data['Fertilidad Infantil (10 a 14 años)'] / pivot_period_data['Afiliadas 10 a 14 años']) * 1000
pivot_period_data['AFR'] = (pivot_period_data['Fertilidad Adolescente (15 a 19 años)'] / pivot_period_data['Afiliadas 15 a 19 años']) * 1000
pivot_period_data['EFR'] = ((pivot_period_data['Fertilidad Infantil (10 a 14 años)'] +
                             pivot_period_data['Fertilidad Adolescente (15 a 19 años)']) / pivot_period_data['Afiliadas 10 a 19 años']) * 1000

pivot_period_data.rename(columns={'Departamento': 'Department'}, inplace=True)

# ==== 3. COMPUTE RELATIVE CHANGES IN FERTILITY RATES (ΔIFR, ΔAFR, ΔEFR) ====
pivot_period_data = pivot_period_data.sort_values(by=["Department", "Año"])
pivot_period_data["ΔIFR"] = pivot_period_data.groupby("Department")["IFR"].pct_change() * 100
pivot_period_data["ΔAFR"] = pivot_period_data.groupby("Department")["AFR"].pct_change() * 100
pivot_period_data["ΔEFR"] = pivot_period_data.groupby("Department")["EFR"].pct_change() * 100

pivot_period_data.dropna(subset=["ΔIFR", "ΔAFR", "ΔEFR"], inplace=True)

# ==== 4. STANDARDIZATION USING Z-SCORE ====
indicators = ["%PIW", "%PMW", "%PSW", "%DVAW", "CHR", "SR"]
for col in indicators:
    pivot_period_data[f"Z_{col}"] = (pivot_period_data[col] - pivot_period_data[col].mean()) / pivot_period_data[col].std()

pivot_period_data["FFI"] = pivot_period_data[[f"Z_{col}" for col in indicators]].mean(axis=1)

ffi_data = pivot_period_data.groupby("Department").last().reset_index()
ffi_data = ffi_data[["Department", "FFI", "ΔIFR", "ΔAFR", "ΔEFR"]].round(2)

# ==== 5. MAP COD_DEPTO FOR PROPER JOIN ====
dep_mapping = {
    "CAUCA": "19", "CHOCO": "27", "ANTIOQUIA": "05", "ATLANTICO": "08", "BOGOTA D.C.": "11",
    "BOYACA": "15", "CALDAS": "17", "CASANARE": "85", "CUNDINAMARCA": "25", "HUILA": "41",
    "META": "50", "QUINDIO": "63", "RISARALDA": "66", "SAN ANDRES": "88", "SANTANDER": "68",
    "TOLIMA": "73", "VALLE": "76", "AMAZONAS": "91", "ARAUCA": "81", "BOLIVAR": "13",
    "CAQUETA": "18", "CESAR": "20", "CORDOBA": "23", "GUAINIA": "94", "GUAVIARE": "95",
    "LA GUAJIRA": "44", "MAGDALENA": "47", "NARINO": "52", "NORTE DE SANTANDER": "54",
    "PUTUMAYO": "86", "SUCRE": "70", "VAUPES": "97", "VICHADA": "99"
}
ffi_data["COD_DEPTO"] = ffi_data["Department"].map(dep_mapping)

# ==== 6. LOAD COLOMBIAN SHAPEFILE ====
shapefile_path = "/content/drive/MyDrive/Sintesis Evidencia/Tadeo/MiTesis/Articulos/Articulo1/Datos/Subregiones_-_Provincias_de_Colombia/Subregiones_-_Provincias_de_Colombia.shp"
colombia_gdf = gpd.read_file(shapefile_path)
colombia_gdf["COD_DEPTO"] = colombia_gdf["COD_DEPTO"].astype(str).str.zfill(2)
colombia_gdf = colombia_gdf.merge(ffi_data, on="COD_DEPTO", how="left")

# ==== 7. PLOT CHOROPLETH MAP AND HEATMAP ====
fig, axes = plt.subplots(1, 2, figsize=(18, 12), gridspec_kw={'width_ratios': [1.5, 1]})

# Choropleth Map with Intervals
bins = [-1.5, -0.5, 0, 0.5, 1.5]
labels = ["< -0.5", "-0.5 - 0", "0 - 0.5", "> 0.5"]
colombia_gdf["EFFI_category"] = pd.cut(colombia_gdf["FFI"], bins=bins, labels=labels)

cmap = mcolors.ListedColormap(["#2166ac", "#67a9cf", "#fddbc7", "#b2182b"])
colombia_gdf.plot(
    column="EFFI_category",
    cmap=cmap,
    ax=axes[0],
    edgecolor="black",
    legend=False
)
axes[0].set_title("Early Fertility Fragility Index (EFFI) by Department", fontsize=14)
axes[0].axis("off")

# Add Custom Legend
legend_labels = ["< -0.5", "-0.5 - 0", "0 - 0.5", "> 0.5"]
legend_colors = ["#2166ac", "#67a9cf", "#fddbc7", "#b2182b"]
for color, label in zip(legend_colors, legend_labels):
    axes[0].scatter([], [], color=color, label=label)
axes[0].legend(title="EFFI Intervals", loc="lower left")

# Heatmap
heatmap_data = ffi_data.sort_values(by="FFI", ascending=False).set_index("Department")[["FFI"]]
sns.heatmap(heatmap_data, cmap="coolwarm", annot=True, fmt=".2f", linewidths=0.5, ax=axes[1])

axes[1].set_title("EFFI Sorted", fontsize=14)
axes[1].set_xlabel("Indicators")
axes[1].set_ylabel("Department")

plt.tight_layout()
plt.show()
heatmap_data
```

Out[37]:

| Attribute | FFI |
| --- | --- |
| Department |  |
| VAUPES | 1.30 |
| ARAUCA | 0.84 |
| LA GUAJIRA | 0.62 |
| GUAINIA | 0.51 |
| AMAZONAS | 0.48 |
| CHOCO | 0.45 |
| VICHADA | 0.26 |
| CAUCA | 0.11 |
| NORTE DE SANTANDER | 0.09 |
| PUTUMAYO | 0.02 |
| SAN ANDRES | -0.01 |
| CESAR | -0.02 |
| NARINO | -0.12 |
| BOLIVAR | -0.13 |
| CAQUETA | -0.14 |
| SUCRE | -0.22 |
| MAGDALENA | -0.26 |
| GUAVIARE | -0.26 |
| CORDOBA | -0.32 |
| CASANARE | -0.32 |
| HUILA | -0.40 |
| ATLANTICO | -0.41 |
| VALLE | -0.45 |
| TOLIMA | -0.49 |
| META | -0.52 |
| QUINDIO | -0.53 |
| ANTIOQUIA | -0.57 |
| CUNDINAMARCA | -0.58 |
| BOYACA | -0.58 |
| CALDAS | -0.59 |
| SANTANDER | -0.60 |
| RISARALDA | -0.62 |
| BOGOTA D.C. | -0.65 |

# Spearman Correlation between ΔIFR, ΔAFR, ΔEFR, and FFI + Scatterplots (Cross validation)¶

In [38]:

```
# Install required libraries if not already installed
!pip install scipy seaborn --quiet

import scipy.stats as stats
import seaborn as sns
import matplotlib.pyplot as plt
import pandas as pd

# ==== 1. STANDARDIZE RELATIVE CHANGES IN FERTILITY RATES (Z-Scores) ====
for delta in ["ΔIFR", "ΔAFR", "ΔEFR"]:
    ffi_data[f"Z_{delta}"] = (ffi_data[delta] - ffi_data[delta].mean()) / ffi_data[delta].std()

# ==== 2. COMPUTE SPEARMAN CORRELATION ====
spearman_results = {}
for delta in ["Z_ΔIFR", "Z_ΔAFR", "Z_ΔEFR"]:
    spearman_corr, p_value = stats.spearmanr(ffi_data["FFI"], ffi_data[delta])
    spearman_results[delta] = (spearman_corr, p_value)

# Convert results into a DataFrame
correlation_df = pd.DataFrame.from_dict(spearman_results, orient="index", columns=["Spearman Correlation", "P-Value"])

# ==== 3. PLOT SCATTER PLOTS WITH TREND LINES & DISPLAY RESULTS IN A 2x2 MATRIX ====
fig, axes = plt.subplots(2, 2, figsize=(18, 12), gridspec_kw={'height_ratios': [1, 1.2]})

# Scatter Plots (Top Row)
for i, delta in enumerate(["Z_ΔIFR", "Z_ΔAFR"]):
    sns.regplot(
        x=ffi_data["FFI"],
        y=ffi_data[delta],
        scatter_kws={"s": 50},
        line_kws={"color": "red"},
        ax=axes[0, i]
    )
    axes[0, i].set_title(f"Scatter Plot: FFI vs. {delta}", fontsize=14)
    axes[0, i].set_xlabel("Fertility Fragility Index (FFI)")
    axes[0, i].set_ylabel(delta)

# Last Scatter Plot (Bottom Left)
sns.regplot(
    x=ffi_data["FFI"],
    y=ffi_data["Z_ΔEFR"],
    scatter_kws={"s": 50},
    line_kws={"color": "red"},
    ax=axes[1, 0]
)
axes[1, 0].set_title(f"Scatter Plot: FFI vs. Z_ΔEFR", fontsize=14)
axes[1, 0].set_xlabel("Fertility Fragility Index (FFI)")
axes[1, 0].set_ylabel("Z_ΔEFR")

# Summary Statistics Table (Bottom Right)
axes[1, 1].axis("off")  # Hide grid
table_data = correlation_df.round(3).reset_index()
table_data.columns = ["Indicator", "Spearman Correlation", "P-Value"]
table = axes[1, 1].table(cellText=table_data.values, colLabels=table_data.columns, cellLoc='center', loc='center')
table.auto_set_font_size(False)
table.set_fontsize(12)
table.scale(1.2, 1.2)  # Resize table

axes[1, 1].set_title("Spearman Correlation Results", fontsize=14, fontweight="bold")

plt.tight_layout()
plt.show()
```

In [17]:

```
table_data
```

Out[17]:

|  | Indicator | Spearman Correlation | P-Value |
| --- | --- | --- | --- |
| 0 | Z\_ΔIFR | 0.330 | 0.061 |
| 1 | Z\_ΔAFR | 0.736 | 0.000 |
| 2 | Z\_ΔEFR | 0.762 | 0.000 |
